# Enantioselectivity of chiral di­hydro­myricetin in multicomponent solid solutions regulated by subtle structural mutation

**DOI:** 10.1107/S2052252523000118

**Published:** 2023-01-21

**Authors:** Jie Sun, Yaoguo Wang, Weiwei Tang, Junbo Gong

**Affiliations:** a School of Chemical Engineering and Technology, State Key Laboratory of Chemical Engineering, The Co-Innovation Center of Chemistry and Chemical Engineering of Tianjin, Tianjin University, Weijin Road, Tianjin 300072, People’s Republic of China; Sun Yat-Sen University, China

**Keywords:** cocrystals, solid solutions, enantiomers, di­hydro­myricetin, caffeine, theophylline

## Abstract

Multicomponent crystals of a chiral drug with non-chiral components have attracted increasing attention in the application of enantiomer purification and regulation of the physicochemical properties of crystalline materials. Crystalline solid solutions provide opportunities for fine-tuning material properties because of continuously adjustable component stoichiometry ratios. The synthesis, crystal structure, thermodynamics and solid-state enantioselectivity of a series of multicomponent crystals of chiral di­hydro­myricetin (DMY) with caffeine (CAF) or theophylline (THE) were investigated and the results reveal how the subtle change of molecular structure of the coformer dictates the enantiomer selectivity in multicomponent cocrystals. A series of multicomponent cocrystal solvates of chiral DMY with CAF and THE were synthesized by the slurry cocrystallization method in aceto­nitrile. Although most racemic mixtures crystallize as racemic compounds or conglomerates, both DMY–CAF and DMY–THE crystallize as chiral solid solutions, unveiled by pseudo-binary melt phase diagrams and pseudo-ternary solution phase diagrams. Crystal structures of *Rac*-DMY–CAF, *R*,*R*-DMY–CAF, *Rac*-DMY–THE and *R*,*R*-DMY–THE are reported for the first time via single-crystal X-ray diffraction, displaying two distinct types of solid solution differing in mixing scale of enantiomers spanning several orders of magnitude. Surprisingly, this remarkable impact on enantiomer discrimination was simply achieved by the reduction of a methyl group of CAF to the THE coformer, which was further rationalized from their crystal structures and intermolecular interactions. Collectively, this work has demonstrated that a subtle change in the molecular structure of a coformer can regulate enantioselectivity in crystalline materials, guiding the purification of chiral racemic compounds via the cocrystallization method and the design of solid-solution crystalline materials.

## Abstract 2

A series of multicomponent cocrystal solvates of chiral di­hydro­myricetin with caffeine (CAF) or theophylline (THE) crystallized as two distinct types of solid solution differing in mixing scale of enantiomers spanning several orders of magnitude. This remarkable impact on enantiomer discrimination was simply achieved by the reduction of a methyl group of CAF to the THE coformer, which was attributed to the hydrogen-bonding donor–acceptor capacity coformers.

## 1. Introduction

In recent years, multicomponent crystals containing a chiral pharmaceutical agent and coformers have provided new opportunities for tuning the physicochemical properties of crystalline materials (Jiang *et al.*, 2021 ▸; Huang *et al.*, 2019 ▸) and for chiral resolution in pharmaceuticals (Guillot *et al.*, 2020 ▸). Chiral compounds generally crystallize into three forms: racemic compounds, conglomerates and solid solutions. More than 95% of chiral compounds crystallize as racemic compounds, roughly 5% exist as conglomerates, and rarely (less than 1%) crystallize as solid solutions (Harfouche *et al.*, 2019 ▸; Rekis, 2020 ▸). Only 25 structures of chiral solid solutions have been reported to date (Rekis *et al.*, 2018 ▸). Despite their rare formation in chiral compounds, solid solutions appear to be prevalent in multicomponent crystals, in which the chiral discrimination or enantioselectivity may be altered by coformer selection (Scowen *et al.*, 2020 ▸; Białońska & Ciunik, 2013 ▸). Few attempts employed this strategy to intentionally design structures that avoid enantioselectivity in the solid state (Friščić *et al.*, 2006 ▸; Chen *et al.*, 2010 ▸; Czapik *et al.*, 2019 ▸; Diniz *et al.*, 2019 ▸). However, the modulation role of the coformer on chiral discrimination or enantioselectivity in multicomponent crystals remains elusive.

Many efforts have been devoted to establishing the relationship between mixed crystals and molecular conformations (Brandel *et al.*, 2013 ▸; Scowen *et al.*, 2020 ▸). From a structural point of view, two types of solid solutions have been suggested in the literature (Chion *et al.*, 1978 ▸). Type I chiral solid solution describes the case where two enantiomers are arbitrarily distributed in the crystal [type I and type II solid solutions suggested by Chion *et al.* (1978); see below Scheme 1(*a*)]. These structures appear to be disordered, with both enantiomers superimposed in asymmetric units; moreover, their occupancy corresponds to the composition of the enantiomers. In a type II chiral solid solution, the crystal structure of the racemic composition is completely ordered. Solid solutions are formed due to the substitution of minor enantiomers by excess enantiomers of essentially the same structure in the non-racemic component.

However, the molecular positions in some solid solutions (Rekis *et al.*, 2018 ▸; Vogt *et al.*, 2010 ▸; Heidi Lopez De Diego *et al.*, 2011 ▸; Esteves De Castro *et al.*, 2007 ▸) of racemic composition are somewhat enantioselective in the formation of racemic components, which are neither completely disordered like in type I, nor completely ordered like in type II. A revised classification criterion [type 1 and type 2 solid solutions; see Scheme 1(*b*)] was proposed by Rekis *et al.* (2018 ▸). The molecular structural features of type 1 solid solutions, *e.g.* thio­camphor (Vogt *et al.*, 2010 ▸; Heidi Lopez De Diego *et al.*, 2011 ▸) and pyrrolidone derivatives (Esteves De Castro *et al.*, 2007 ▸), were further elucidated based on type I. There is a statistical mirror plane intersecting the molecule [Scheme 1(*b*)] which thus generates the opposite enantiomer within the same molecular site. The single enantiomeric crystal of such solid solutions has an asymmetric unit molecule number *Z*′ = 1, whereas the crystal structure with racemic components has an asymmetric unit molecule number *Z*′ = 0.5. However, in type 2 solid solutions (Esteves De Castro *et al.*, 2007 ▸, Rekis *et al.*; 2018 ▸; Baert *et al.*, 1978 ▸; Kroon *et al.*, 1976 ▸; Rekis & d’Agostino *et al.*, 2017 ▸; Rekis & Bērziņš *et al.*, 2018 ▸), the number of molecules in the asymmetric unit of a single enantiomer crystal is often even, and molecular conformational adjustment allows a reasonable pseudo-inversion center between two or an even number of molecular conformations in the asymmetric unit. This enantiopure structure mimics a centrosymmetric one by adjusting the molecular conformations so a reasonable pseudo-centrosymmetry might be attained between *R*I and *R*II. When some amount of the opposite enantiomer *S* is introduced, it can adopt conformation I and occupy the former *R*II sites in the crystal structure. Thus, locally there is genuine centrosymmetry between *R*I and *S*I present.[Chem scheme1] 
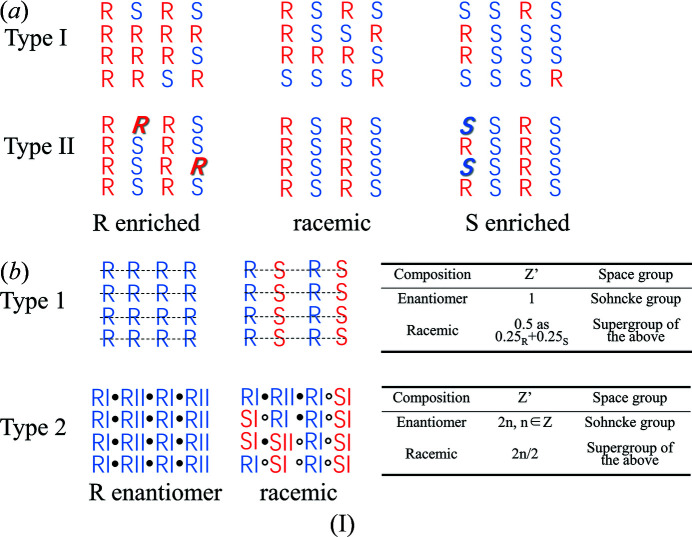


DMY is the main flavanol compound isolated from a traditional Chinese medicine ampelopsis grossedentata, which shows hepatoprotective, antioxidative, anti-inflammatory and antihypertensive effects (Liu *et al.*, 2019 ▸). DMY has two chiral centers and thus has four potential enantiomers (Lin *et al.*, 2019 ▸). Note that *Rac*-DMY crystallizes as a racemic compound containing *R*,*R*-DMY and *S*,*S*-DMY in the asymmetric unit (Xu *et al.*, 2007 ▸), nonetheless, homochiral *R*,*R*-DMY showed higher anti-inflammatory activity than *Rac*-DMY (Wang *et al.*, 2016 ▸). In this work, we used DMY as a chiral model substance to examine the role of the molecular structure of the coformer on chiral discrimination in multicomponent crystals from the perspectives of crystal structure, thermodynamics and intermolecular interactions. Two cocrystal coformers, caffeine (CAF) and theophylline (THE), were selected with a subtle difference in molecular structure. The crystal structures of *R*,*R*-DMY–THE, *Rac*-DMY–THE, *R*,*R*-DMY–CAF and *Rac*-DMY–CAF cocrystal solvates are, to our knowledge, reported for the first time, revealing the two types of solid solutions constituting chiral cocrystals that display several orders of magnitude mixing performance of two enantiomers within the crystals. The distinct enantioselectivity difference in the two chiral cocrystal systems can be attributed to the hydrogen-bonding donor–acceptor capacity of the coformers.

## 2. Materials and methods

### 2.1. Materials

Racemic DMY (>99.9%, GC) and *R*,*R*-DMY (>99.9%, GC) were purchased from Hangzhou Lin Ran Biotech Co. Ltd. Aceto­nitrile (purity >99.9%, GC) was purchased from Concord Technology Co. Ltd. CAF and THE of purity >99.0% (GC) were purchased from Aladdin (Shanghai) Bio-Chem Technology Co. Ltd. All chemicals were used as received.

### 2.2. Preparation and characterization of cocrystals

A suspension of 0.1 mmol (32 mg) DMY with 0.1 mmol CAF (19.4 mg) or 0.1 mmol THE (18 mg) and 2 ml aceto­nitrile was prepared and stirred at 30°C for 24 h. The cocrystals obtained were filtered from the suspension, and the supernatant was slowly volatilized at room temperature to prepare single crystals of suitable size that were structurally resolved using single-crystal X-ray diffraction (SCXRD).

Powder X-ray diffraction (PXRD) was performed on a D/MAX 2500 diffractometer at 40 kV and 100 mA coupled with Cu *K*α radiation (λ = 1.5406 Å). The samples were scanned over the 2θ range 4−40 ° at a speed of 8° min^−1^.

Scanning electron microscopy (SEM) was used to observe the twin crystal structure of the *Rac*-DMY–THE samples. All samples were observed on a TESCAN MIRA LMS scanning electron microscope operated under a vacuum with an accelerating voltage of 15.00 kV.

### 2.3. Construction of the binary melt phase diagram

A series of known enantiomeric compositions of DMY powders were completely dissolved in aceto­nitrile with equimolar THE at 50°C. The clear solution was slowly cooled to 25°C and stirred for 8 h to prepare DMY–THE cocrystals with a uniform size distribution. The harvested crystals were characterized by PXRD to verify the cocrystal formation. The samples were dried at 50°C for 12 h and characterized by differential scanning calorimetry (DSC), thermogravimetric analysis (TGA) and hot-stage microscopy (HSM) to determine the melting properties.

DSC was performed on a Mettler Toledo DSC 1 STARe system under a nitro­gen atmosphere. Approximately 6 mg of sample was added to a standard DSC aluminium pan and heated at a rate of 10°C min^−1^. The onset of the endotherm was chosen as the melting temperature for the construction of the binary phase diagram.

TGA experiments were conducted on a Mettler TGA/DSC 1 STARe System. Approximately 6.0 mg of the sample was heated from 30 to 200°C at 10 °C min^−1^ under a nitro­gen flow (50 ml min^−1^).

HSM studies were performed on an Olympus BX-51 microscope equipped with a DSC600 hot stage Linkam system.

### 2.4. Measurement of the ternary phase diagram

The ternary phase diagram of the DMY–THE cocrystal was constructed in aceto­nitrile at temperatures ranging from 25 to 45°C. Known proportions of racemic and *R*,*R*-DMY mixture as well as THE powders were added to a crystallizer with approximately 30 ml aceto­nitrile at a given temperature. The suspension was stirred at a speed of 300 rpm. The crystalline phase of solid samples was monitored by PXRD for 30 min to evaluate the status of the solid–liquid equilibrium at a given temperature. The equilibrium time on solubility measurements was determined by measuring the concentration and components of the extracted clear supernatant using chiral high-performance liquid chromatography (HPLC) every 24 h until the concentration no longer varied. The preliminary tests provide an equilibrium time of 120 h. The suspended solid samples were subjected to PXRD analysis for the determination of the crystalline phase. The saturated liquid phase was suitably diluted before concentration measurements using chiral HPLC. The measurement at each condition was repeated three times to ensure the reliability of experimental results. Finally, the ternary phase diagram was constructed from the determined masses of DMY, THE and the solvent.

Solubility and solution concentration were determined using chiral HPLC [column: Phenomenex Lux Amylose-1, 250 mm × 4.6 mm × 5 µm; eluent: methanol and water with 0.1% tri­fluoro­acetic acid (2:8 *v*/*v*); flow rate 0.4 ml min^−1^; injection volume 10 µl; column temperature 25 °C; retention time 30 min]. The sampling of 0.1–0.2 ml solution was performed using a syringe and a syringe filter (0.22 µm pore size). The calibration curves of *S*,*S*-DMY, *R*,*R*-DMY and THE show a good linear fit with *R* ^2^ > 0.999 (Fig. S9).

### 2.5. Single-crystal X-ray diffraction

SCXRD data were collected on an Agilent Rigaku Super Nova diffractometer with a CCD detector system. Suitable crystals of appropriate dimensions were chosen and mounted on loops for data collection. Preliminary unit-cell parameters were obtained with three sets of twelve narrow frame scans. The *CrysAlisPro* data reduction package (Rigaku, 2019 ▸) was used for acquisition, indexing, integration, absorption correction and scaling of Bragg reflections. The final cell parameters were determined from all reflection data. A Gaussian face-indexed absorption correction was applied to the three datasets collected. *CrysAlisPro* was also used for the analysis of systematic absences and space group determination. Thereafter, the *OLEX2* software (Dolomanov *et al.*, 2009 ▸) was used to the solve crystal structure by direct methods (*SHELXT*; Sheldrick, 2015 ▸) and refined by full-matrix least-squares on *F* ^2^ for all data (*SHELXL*; Sheldrick, 2008 ▸; Parsons *et al.*, 2013 ▸). The non-hydrogen atoms were refined anisotropically. All hydrogen atoms were located from electron-density difference maps and were positioned geometrically and refined using the riding model [*U*_iso_(H) = 1.2*U*_eq_ or 1.5*U*_eq_]. The occupation of parts attributed to the enantiomers in the crystal were refined freely. *Mercury* (version 3.10; Macrae *et al.*, 2020 ▸) was used for structure analysis and visualization.

### 2.6. Selective dissolution experiments of the *Rac*-DMY–THE cocrystal

*Rac*-DMY–THE cocrystals grown from a racemic solution were selected and placed in a saturated solution of *R*,*R*-DMY–THE for selective dissolution experiments. When *Rac*-DMY–THE cocrystals crystallized as epitaxial conglomerates or inversion twins, the racemic cocrystals are expected to partially dissolve. A polarizing optical microscope (Zeiss Primotech) was used to monitor the dissolution of the *Rac*-DMY–THE cocrystals over time.

### 2.7. Hirshfeld surface and 2D fingerprint plots

The Hirshfeld surface (HS) and 2D fingerprint plots were generated using the *Crystal Explorer* software package (version 3.148; Spackman *et al.*, 2021 ▸) with the experimental SCXRD data as input. The color pattern on the HS shows the areas involved in interactions. On the *d*_norm_ surfaces, the red, white and blue areas represent the atomic contacts shorter than, equal to and longer than the sum of their van der Waals radii, respectively.

## 3. Results and discussion

### 3.1. Characterization of racemic and enantiomorph cocrystals

**Figure 1:**
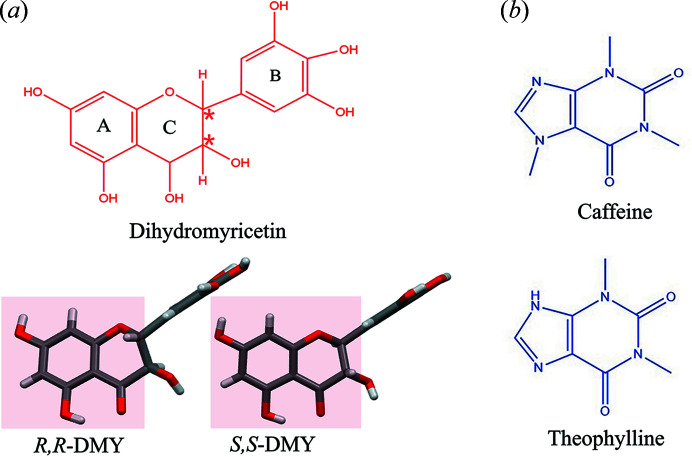
Molecular structure of (*a*) DMY and the two selected coformers (*b*) CAF and THE. The two chiral carbons are highlighted in red stars with the two predominant enantiomers of DMY in solution (coplanar A and C six-membered rings are highlighted in light red).

Two chiral carbons exist in the molecular structure of DMY [Fig. 1 ▸(*a*)] which constitute two coplanar six-membered rings (highlighted in red) A and C, and another perpendicular plane of the six-membered ring B. The hydrogen atom at each chiral carbon position is located on the same side of the coplane of the A and C rings. The structural characteristics produce a total of four potential enantiomers in which *S*,*S*-DMY and *R*,*R*-DMY are the dominant enantiomers in solution and crystallize to be *Rac*-DMY dihydrate from aqueous solution (Wang *et al.*, 2016 ▸).

**Figure 2:**
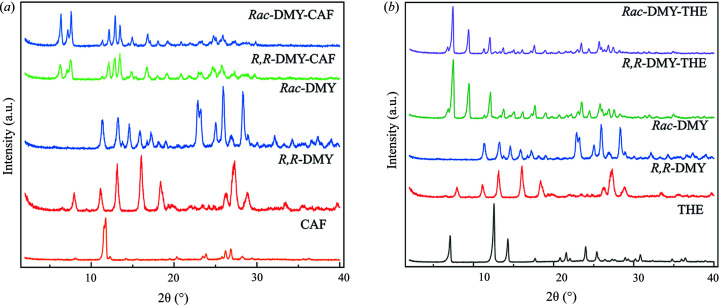
(*a*) PXRD patterns of *Rac*-DMY–CAF and *R*,*R*-DMY–CAF in comparison with *Rac*-DMY, *R*,*R*-DMY and CAF. (*b*) PXRD patterns of *Rac*-DMY–THE and *R*,*R*-DMY–THE in comparison with *Rac*-DMY, *R*,*R*-DMY and THE.

The suspension crystallization of *Rac*-DMY or *R*,*R*-DMY with CAF powders in aceto­nitrile results in the formation of a new phase. As shown in Fig. 2 ▸(*a*), the PXRD pattern of *Rac*-DMY–CAF displays several characteristic peaks located at 6.5, 7.4, 7.8, 12.3, 13.0 and 13.7°, differing from that of DMY or CAF crystals. Further, the experimental PXRD pattern matches well with the calculated one derived from the solved crystal structure (Fig. S1 in supporting information). These results corroborate the formation of the *Rac*-DMY–CAF cocrystal. Besides, the characteristic peaks of the *R*,*R*-DMY–CAF phase are identical to those of *Rac*-DMY–CAF cocrystals, indicating the formation of either a conglomerate of enantiomorph cocrystals or a chiral solid solution.

In contrast to CAF, the structure of THE coformer only lacks a methyl group on the five-membered ring, as shown in Fig. 1 ▸(*b*). This structural nuance was previously used to probe the chiral discrimination in cocrystal formation (Friščić *et al.*, 2006 ▸). Here, we observed that *R*,*R*-DMY–THE and *Rac*-DMY–THE exhibit identical PXRD patterns that are different from the DMY or THE samples [Fig. 2 ▸(*b*)], suggesting the formation of a new crystalline phase. PXRD patterns of these samples are identical to the calculated ones simulated from the solved crystal structure (Fig. S2), corroborating the formation of *R*,*R*-DMY–THE and *Rac*-DMY–THE cocrystals.

### 3.2. Crystal structure analysis

#### 3.2.1. *Rac*-DMY–CAF and *R*,*R*-DMY–CAF cocrystals

Further crystallographic structure determinations of *Rac*-DMY–CAF and *R*,*R*-DMY–CAF cocrystals were performed to differentiate conglomerates or solid solutions of enantiomorph cocrystals. Single crystals of sufficient size and mass were grown from aceto­nitrile solution to perform SCXRD analysis. Owing to the presence of compositional disorder of the racemic component (*i.e.* solid solution), Ga *K*α radiation was employed to collect crystallographic data of *Rac*-DMY–CAF and *R*,*R*-DMY–CAF cocrystals. The high density of disorder/defects in the crystal structure may result from the wrong sites occupied by enantiomers in the structure, generating high *R*_1_ values (Table 1 ▸).

**Figure 3:**
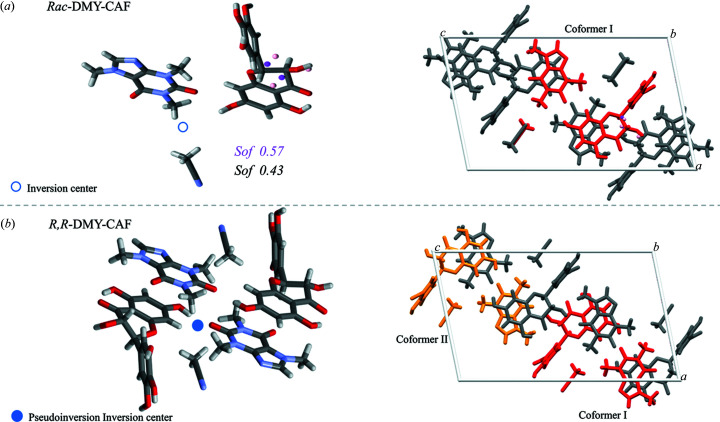
Asymmetric units and unit cells of (*a*) *Rac*-DMY–CAF (*P*2_1_/*n*, *Z*′ = 1, *Z*′′ = 3) and (*b*) *R*,*R*-DMY–CAF (*P*2_1_, *Z*′ = 2, *Z*′′ = 6).

The *Rac*-DMY–CAF cocrystal crystallizes in the monoclinic space group *P*2_1_/*n*. The asymmetric unit is composed of equimolar amounts of DMY, CAF and aceto­nitrile, as illustrated in Fig. 3 ▸. The analyses of the anisotropic shift ellipsoid and the differential electron density map confirm the inclusion of a disordering coformer in the crystal structure and the proportion of disorder confirmed is 0.43–0.57. We thus conclude that *Rac*-DMY–CAF does not belong to the typical racemic compound crystallizing system.

**Figure 4:**
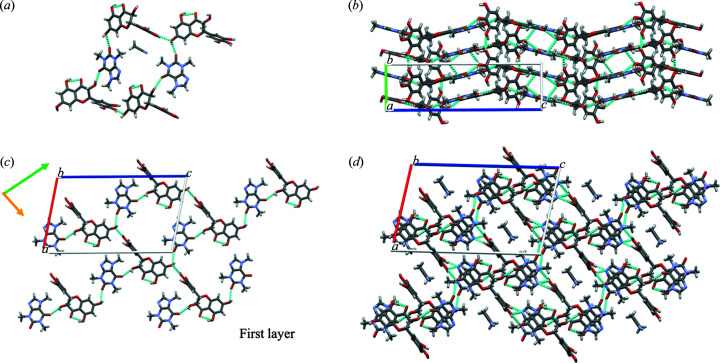
Structure analysis of the *Rac*-DMY–CAF cocrystal. (*a*) Hydrogen-bonding pattern showing the formation of a channel structure with aceto­nitrile included. (*b*) 3D molecular packing arrangements between DMY and CAF molecules along the *b* direction. (*c*) Hydrogen-bonding arrangement and (*d*) 3D molecular packing with an aceto­nitrile molecule included, viewed along the *b* direction.

As shown in Fig. 4 ▸(*a*), two types of intermolecular hydrogen bonds are observed in the crystal structure: an O—H⋯O—H hydrogen bond is formed between hydroxyl groups of DMY molecules, and an O—H⋯O=C hydrogen bond is formed between the hydroxyl group of the DMY molecule and the carbonyl group of the CAF molecule. DMY and CAF form long zigzag chains that constitute alternating layers perpendicular to the *b* axis [Fig. 4 ▸(*b*)]. In each layer, the hydroxyl group on the DMY chiral C center passes through the O—H⋯O=C hydrogen bond (2.635, 1.90, θ: 144.8) with the carbonyl group of CAF (green arrow), and the hydroxyl group of DMY resorcinol connects with another carbonyl group of CAF via an O—H⋯O=C hydrogen bond (2.652, 2.29, θ: 106.0). In the perpendicular direction (orange arrow), the hydroxyl group of DMY resorcinol links to the adjacent catechol of DMY via an O—H⋯H—O hydrogen bond (2.769, 2.15, θ: 133.0). These layers were stacked by an O—H⋯N hydrogen bond (2.716,1.90, θ: 164.1) to form a 3D network with aceto­nitrile molecules included in the channel. Detailed data on hydrogen bonding interactions are shown in Table S2. The single crystal of the *Rac*-DMY–CAF cocrystal obtained through the slow volatilization of the solvent contains a disordering coformer with a proportion of 0.57. However, the crystal structure is largely (but not completely) an ordered arrangement in terms of enantiomer layout.

In previous studies, it was suggested that the functional groups near the chiral center do not participate in hydrogen bonding interactions in the crystal structure of the solid solution forming system, to assure the replacement of the conformation of the enantiomer (Rekis & d’Agostino *et al.*, 2017 ▸; Rekis *et al.*, 2017 ▸). But in this case, we observed the direct formation of hydrogen bonding interactions of the hydroxyl group or ophthalmic triol group of DMY at the chiral carbon center with adjacent molecules. Interestingly, this packing arrangement will not be altered when enantiomer replacement occurs within the crystal structure, leading to the formation of *Rac*-DMY–CAF cocrystal solid solution.

The *R*,*R*-DMY–CAF cocrystal crystallizes in the monoclinic space group *P*2_1_. As shown in Fig. 3 ▸(*b*), the asymmetric unit of *R*,*R*-DMY–CAF contains two sets of equimolar DMY, CAF and aceto­nitrile molecules. The anisotropic displacement parameters (ADPs) do not show any disorder in the *R*,*R*-DMY–CAF crystal structure. In general, the rigidity and stable stacking of molecules limit the possibility of molecular disorder encountered in solid solutions. The packing arrangments of the *R*,*R*-DMY–CAF and *Rac*-DMY–CAF cocrystals are almost identical, which explains their similar PXRD patterns [Fig. 2 ▸(*a*)].

**Figure 5:**
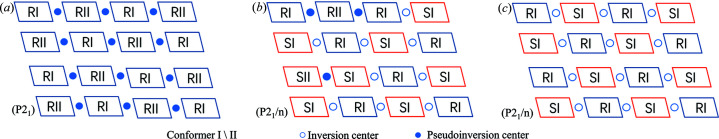
Graphical representation of the solid solution formation of the *Rac*-DMY–CAF cocrystal structure. (*a*) Enantiopure composition (note that the depicted inversion centers are not genuine, but only indicate a pseudosymmetry between coformers *R*I and *R*II). (*b*) Racemic composition as observed for most experimentally determined structures (statistical inversion centers). (*c*) Racemic composition as proposed by Chion *et al.* (1978 ▸) (genuine inversion centers).

The determination of the space group *P*2_1_ in the crystal structure of *R*,*R*-DMY–CAF rather than *P*2_1_/*n* is based on *R*_1_ and *R*_int_ values. Electron statistical analysis suggests the presence of centrosymmetric space groups which can be explained by the pseudosymmetry of the crystal structure, illustrated in Fig. 5 ▸. The true centrosymmetry of the racemic composition can be achieved by the pairs *S*I–*R*I or *S*II–*R*II. For enantiomerically pure samples, two groups of molecules with homochirality can adjust the conformation to form a centrosymmetric pair, so that the formation of a quasi-centrosymmetric structure is also able to host the opposite enantiomer when the scalar composition phase was considered. The requirement for a solid solution is that the two (or another even number of) enantiomeric molecules in the asymmetric unit mirror are approximately mirror images of each other. The crystal structure of the DMY–CAF cocrystal meets all structural prerequisites to be the type 2 solid solution; moreover, the crystal structure of *R*,*R*-DMY–CAF is similar to that of *Rac*-DMY–CAF (see Fig. 3 ▸). The structural origin of such type of solid solution formation could be presumably due to non-racemic mixtures forming quasi-centrosymmetric structures, in which the number of missing enantiomers is compensated by the adoption of enriched enantiomers in a conformation similar to that of the minority enantiomer. This phenomenon can be referred to as shape mimicry (Fayzullin *et al.*, 2017 ▸). Thus, the enantiomerically pure and racemic structures are isomeric, and continuous changes in their enantiomeric composition may form identical isomeric stacks. For conformationally flexible molecules, the stability of the crystal structure usually depends on a subtle compromise between the intermolecular interaction energy and the stacking efficiency. Overall, the DMY–CAF cocrystal system conforms to the crystallographically pseudo-centrosymmetric mixed-phase structure of typical type 2 solid solutions as classified by Rekis & Bērziņš (2018 ▸).

#### 3.2.2. *Rac*-DMY–THE and *R*,*R*-DMY–THE cocrystals

The SCXRD data of *R*,*R*-DMY–THE were collected using Mo *K*α radiation, and the conformation of *R*,*R*-DMY was determined by chiral HPLC as its chiral conformation remains stable during the formation of cocrystals (Wang *et al.*, 2016 ▸). The *R*,*R*-DMY–THE cocrystal crystallizes in an orthorhombic crystal system with the space group *P*2_1_2_1_2_1_, and the crystallographic data are shown in Table 2 ▸. The asymmetric unit of the cocrystal consists of THE, *R*,*R*-DMY and aceto­nitrile in the molar ratio 1:1:1, and there is no disorder in the crystal structure.

**Figure 6:**
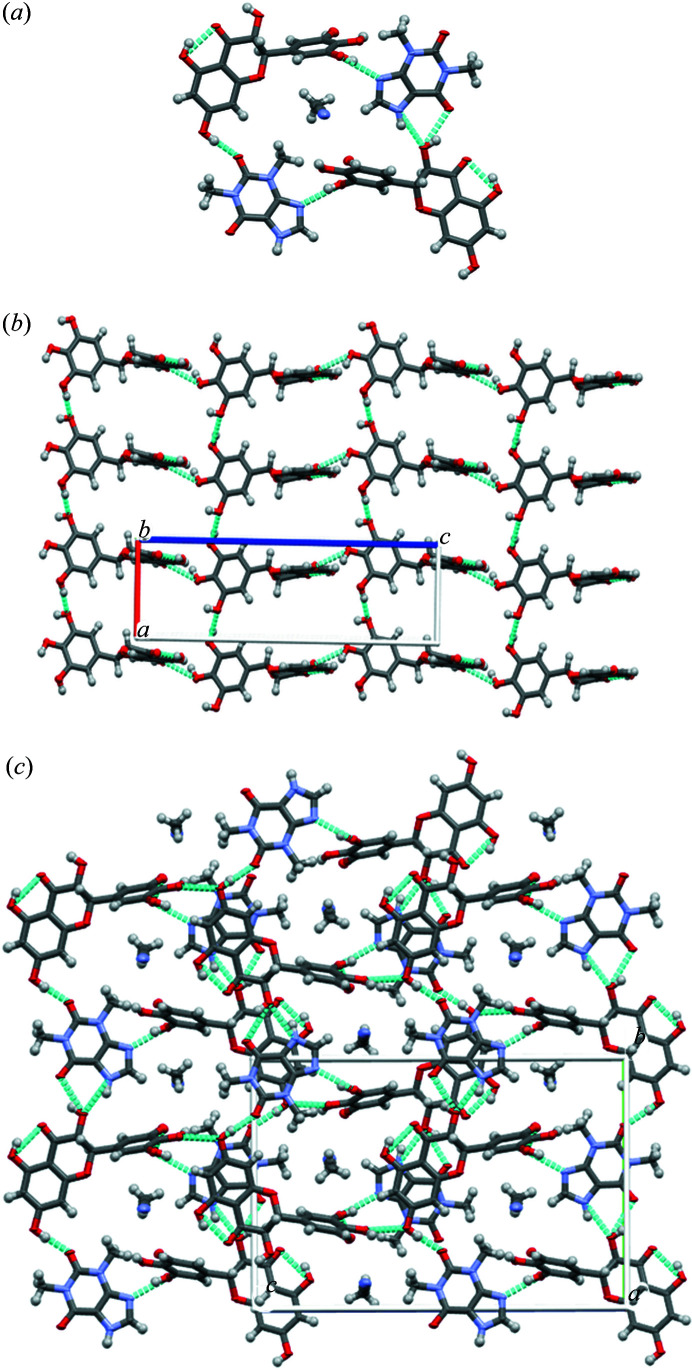
Structure analysis of the *R*,*R*-DMY–THE cocrystal. (*a*) Hydrogen-bonding pattern showing the formation of the channel structure with aceto­nitrile included. (*b*) 3D molecular packing arrangement between pyrogallol–pyrogallol along the *a* direction and hydrogen bonding arrangement between resorcinol–pyrogallol along the *c* direction; (*c*) 3D molecular packing with an aceto­nitrile molecule included, viewed along the *a* direction.

In the crystal structure, *R*,*R*-DMY interacts with two THE molecules by forming O—H⋯N and O—H⋯O hydrogen bonds, where one THE molecule interacts with *R*,*R*-DMY via O—H⋯N (2.764, 1.95, θ: 162.7), and the other THE also forms hydrogen bonds with the second *R*,*R*-DMY via O—H⋯O (2.720, 2.00, θ: 142.6) and N—H⋯O (2.738, 1.97, θ: 145.3), forming channels in the crystal structure to accommodate the aceto­nitrile guest molecule [Fig. 6 ▸(*a*)]. The adjacent *R*,*R*-DMY molecules interact with each other by forming O—H⋯O hydrogen bonds (2.7203, 1.9, θ: 156.1; 2.834, 2.27, θ: 125.1) between pyrogallol rings along the *a* direction and between the pyrogallol ring (B ring) and resorcinol ring (A ring) along the *c* direction [Fig. 6 ▸(*b*)], which results in a 3D host network [Fig. 6 ▸(*c*)]. The aceto­nitrile guest molecule is included within the host network through weak C—H⋯N hydrogen bonding interactions. Detailed hydrogen bonding interaction data are shown in Table S3. Unlike the structure of the *R*,*R*-DMY–CAF co-crystal, the asymmetric unit of the *R*,*R*-DMY–THE co-crystal has only one set of THE, *R*,*R*-DMY and aceto­nitrile molecules and does not form another set of conformational molecules to mimic the conformations of THE, *S*,*S*-DMY and aceto­nitrile in the racemic structure. Therefore, it does not conform to the typical crystallographic pseudo-centrosymmetric mixed-phase structural features of type 2 solid solutions.

**Figure 7:**
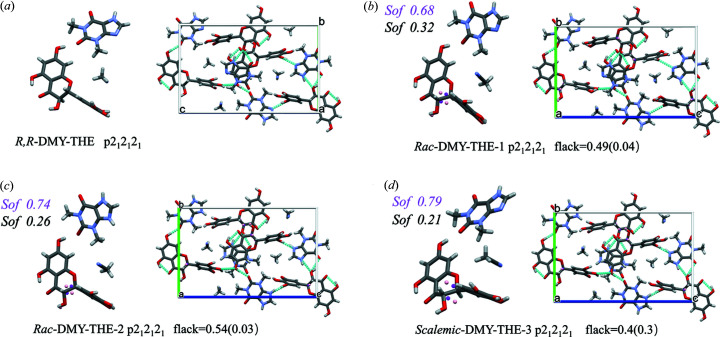
Asymmetric units and unit cells of *R*,*R*-DMY–THE (*P*2_1_2_1_2_1_, *Z*′ = 1, *Z*′′ = 3), *Rac*-DMY–THE (*P*2_1_2_1_2_1_, *Z*′ = 1, *Z*′′ = 3) and *Scalemic*-DMY–THE (*P*2_1_2_1_2_1_, *Z*′ = 1, *Z*′′ = 3).

Single crystals of the *Rac*-DMY–THE cocrystal were obtained by slow volatilization from a 1:1 solution of the racemic composition of DMY and THE in aceto­nitrile. Single crystals of suitable size and relatively good quality were selected, and crystallographic data were collected using a Cu target to receive information on reliable chiral conformations. Single-crystal structure determination shows that both enantiomers crystallized in the orthorhombic Sohncke space group *P*2_1_2_1_2_1_. The asymmetric units contain equimolar amounts of THE, DMY and aceto­nitrile (Fig. 7 ▸). Crystal packing analysis reveals the identical molecular conformation and packing arrangements between *Rac*-DMY–THE-1 (or *Rac*-DMY–THE-2) (Table 2 ▸) and *R*,*R*-DMY–THE cocrystals. This likely indicates that the *Rac*-DMY–THE cocrystal is a conglomerate, but the analyses of anisotropic shift ellipsoids and differential electron density maps show the presence of disordered molecules. To demonstrate the lack of enantioselectivity in the solid state, the structure of the third scalar crystal (*Scalemic*-DMY–THE-3) was also determined, unveiling the disordered nature of scalemic crystals. The Flack values of *Rac*-DMY–THE-1 and *Rac*-DMY–THE-2 cocrystals are equal to 0.49(0.04) and 0.54(0.03), respectively, which, given plausible uncertainties, indicate that single crystals grown in saturated solutions of racemization are racemic twins, with domains of opposite absolute structure, statistically mosaic in the same crystal. The ratio of two absolute structured crystal domains can be determined by the Flack value. The disorder percentages in *Rac*-DMY–THE-1 and *Rac*-DMY–THE-2 cocrystals are 0.68 and 0.74, respectively. In each case (as long as the phases are assumed to be thermodynamically equilibrated), the exact degree of disorder should be related to the conformational entropy, which is determined by several isoenergetic local structure models. Disorder in crystal structures of other types of molecules has been explained and is thought to be controlled by Boltzmann statistics (Rekis, 2019 ▸). The degree of disorder and the conformational ratio or molecular positional enantioselectivity should be directly related to the energetic aspects of the intermolecular interactions (Zhang *et al.*, 2017 ▸; Vogt *et al.*, 2010 ▸ *b*). The Flack value for *Scalemic*-DMY–THE-3 is 0.4 with an *R*_1_ value of 3.55%, but the uncertainty of 0.3 is too large. The lattice parameters and molecular volumes of the cocrystals are shown in Table 2 ▸. There is no significant enantiomeric composition-dependent variation in the cell parameters, unit-cell volume and crystal density. The cell parameters (*i.e.* *a*, *b* and *c*) of the racemic samples are slightly increased compared with the enantiomerically pure crystals, which implies a slight expansion of the cell volume because of the disorder, although the experimental temperature may increase the cell parameters. We thus concluded that either the racemic or the enantiomerically pure sample of the DMY–THE cocrystal can form a uniformly stacked crystal structure without crystallization into a true conglomerate. Furthermore, the *Rac*-DMY–THE cocrystal does not belong to the type 1 solid solution, which requires the number of molecules in the asymmetric unit of the racemate crystal to be half that of a single enantiomer crystal (Rekis & Bērziņš, 2018 ▸).

### 3.3. Thermodynamics of *Rac*-DMY–THE cocrystals

Racemic twins that crystallize in the Sohncke space group are a different type of conglomerate in which each crystal contains two enantiomers in separate structural domains, each containing only one of the two enantiomers. There are several reports of epitaxially grown conglomerates that can display racemic composition (Kaptein *et al.*, 2008 ▸; Davey *et al.*, 2014 ▸; Spix *et al.*, 2014 ▸). In solid solution, although the two enantiomers are present in equal numbers, they are randomly distributed in the structure (irregular enantiomeric arrangement) and have no longevity. Solid solutions can crystallize in non-Sohncke or Sohncke space groups. Because of different scales of enantiomeric distribution, we explored the thermodynamic characteristics of racemic twinned crystals of the *Rac*-DMY–THE cocrystal in this section.

#### 3.3.1. Pseudo-binary melt phase diagram of *Rac*-DMY–THE cocrystals

**Figure 8:**
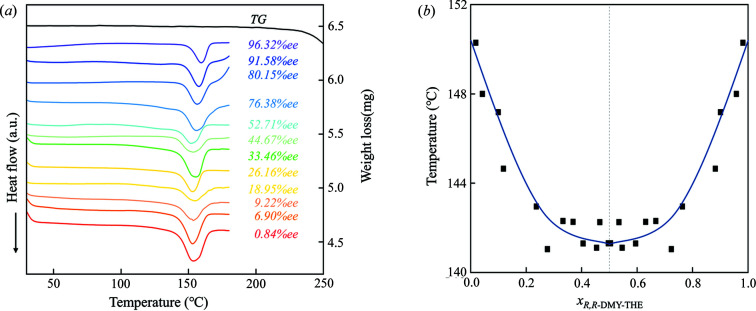
(*a*) TGA-DSC curves for the DMY–THE cocrystals at various *ee*. (*b*) Pseudo-binary melt phase diagram of DMY–THE cocrystals.

A series of DMY–THE cocrystals composed of different chiral DMY enantiomers were prepared for DSC analysis. As shown in Fig. 8 ▸(*a*), there is no weight loss observed in cocrystal samples until about 180°C. This is further confirmed by temperature-variant hot-stage microscopy presented in Figs. S3–S4 of the supporting information. The observed endothermic peak around 140–150°C can be attributed to the melting of cocrystal samples. The onset temperature is considered to be the melting point of the samples, and the measured data are given in Table S1 of the supporting information. We found that each cocrystal mixture, regardless of enantiomeric composition, has only one broad endothermic peak, indicative of chiral solid solutions. Further, the melting point decreases monotonically with the increase of *S*,*S*-DMY in the cocrystal with the maximum at 150.29°C for the 96.32%*ee* DMY–THE cocrystal mixtures and the minimum at 141.31°C for the 0.84%*ee* DMY–THE sample. These measured melting point data can be used to construct a Pseudo-binary melt phase diagram [Fig. 8 ▸(*b*)]. The *Rac*-DMY–THE cocrystal is a continuous solid solution exhibiting the lowest melting point at the racemic composition.

#### 3.3.2. Pseudo-ternary solution phase diagram of *Rac*-DMY–THE cocrystals

**Figure 9:**
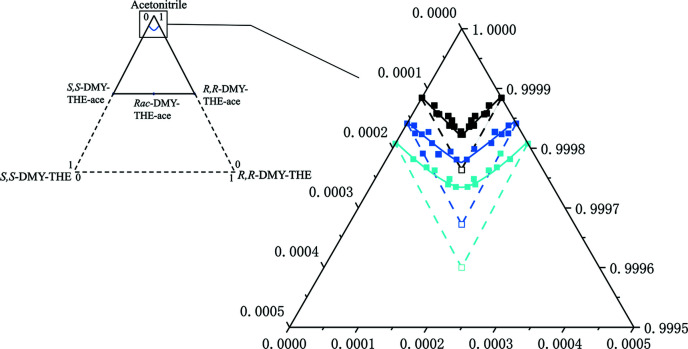
Pseudo-ternary solution phase diagram of *Rac*-DMY–THE cocrystals in aceto­nitrile.

Solution ternary phase diagrams are useful for distinguishing the crystalline form of a chiral compound and can provide the most basic and reliable data for chiral resolution (Srisanga & Horst, 2010 ▸). The solubility data of a series of DMY–THE cocrystal mixtures composed of different enantiomeric compositions in aceto­nitrile were measured at 25, 35 and 40 °C. Solid–liquid phase equilibrium experiments were performed, and the concentration of the clear supernatant was measured with an interval of 24 h by chiral HPLC to determine the solubility of the DMY–THE cocrystal mixtures of different enantiomeric compositions. The solid phase was subjected to PXRD analysis to rule out any phase transformation throughout the experiments. We found that the pseudo-ternary phase diagram reaches an equilibrium state after 120 h. Fig. 9 ▸ shows the established pseudo-ternary phase diagram, and the colored lines represent the solid–liquid equilibrium lines at different temperatures. The solubility of DMY–THE cocrystals increases with the rise in temperature and the decreases of *R*,*R*-DMY enantiomer in the solid phase. The solubility curves conform to the shape of a type of continuous solid solution, which is consistent with the results of the pseudo-binary melt phase diagram that displays a downward-concave shape. When the enantiomer system crystallized as a conglomerate or lamellar conglomerates (van Enckevort, 2010 ▸; Zlokazov & Pivnitsky, 2020 ▸), the solubility of the crystal mixtures composed of equimolar enantiomers should be twice the solubility of the single enantiomer crystal, conforming to the ‘Meyerhoffer’ double solubility rule (Izumi & Blackmond, 2009 ▸). The colored hollow squares represent the eutectic point when the solubility of the racemic co-crystal is twice the solubility of an enantiomeric co-crystal. However, a closer look at the lowest point of the curve shows that the measured solubility of the DMY–THE cocrystals composed of equimolar enantiomers is much smaller than the expected value of the double solubility curve. An epitaxial lamellar conglomerate or multilayered conglomerate displaying single enantiomeric crystals crystallized in a chiral space group but in different chiral compositions, even with no enantiomeric excess, has been reported in the literature and is similar to our case. By combining the determined binary melt phase diagram and the ternary solution phase diagram, the chiral DMY–THE cocrystal system is considered to be a type of continuous solid solution.

#### 3.3.3. Selective dissolution of enantiomers

**Figure 10:**
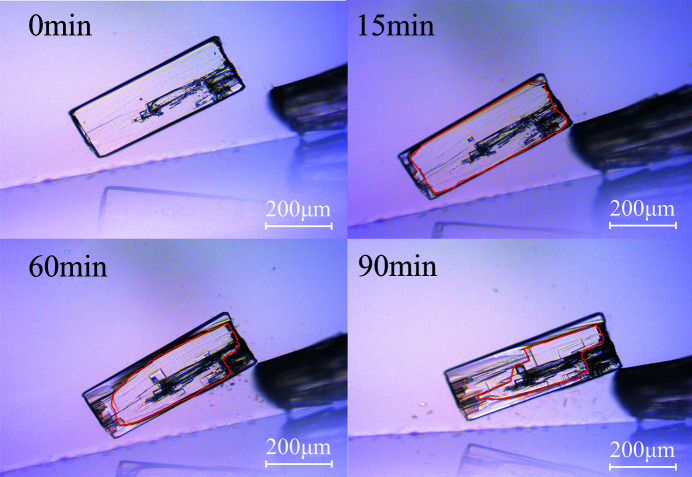
Time-resolved optical micrographs of the *Rac*-DMY–THE cocrystal in a saturated *R*,*R*-DMY–THE solution showing the selective dissolution of crystal fragments.

We further employed selective dissolution experiments to understand the mixing scale of enantiomeric cocrystals in a solid solution of DMY–THE, which is not suggested on the whole crystal scale nor on the molecular scale. The selective dissolution experiments were previously used in epitaxially grown conglomerate-forming systems to probe the distribution of enantiomeric domains in the crystal (Kaptein *et al.*, 2008 ▸; van Eupen *et al.*, 2008 ▸). When the *Rac*-DMY–THE cocrystal was placed in a saturated enantiomerically pure solution, we observed the enantioselective dissolution of crystal fragments under an optical microscope (Fig. 10 ▸), which suggests the presence of enantiomeric crystal domains. The *Rac*-DMY–THE cocrystal is composed of many *R*,*R* and *S*,*S* enantiomeric domains but displays near-racemic domains. Moreover, a small flake was peeled off the crystal of the racemate by the needle and measured by chiral HPLC which shows an enantiomeric excess value of about 79.8% (Fig. S7) and thus confirms the spatial heterogeneity of enantiomer domains in the solid solution of the *Rac*-DMY–THE cocrystal.

#### 3.3.4. Observation of racemic twins

**Figure 11:**
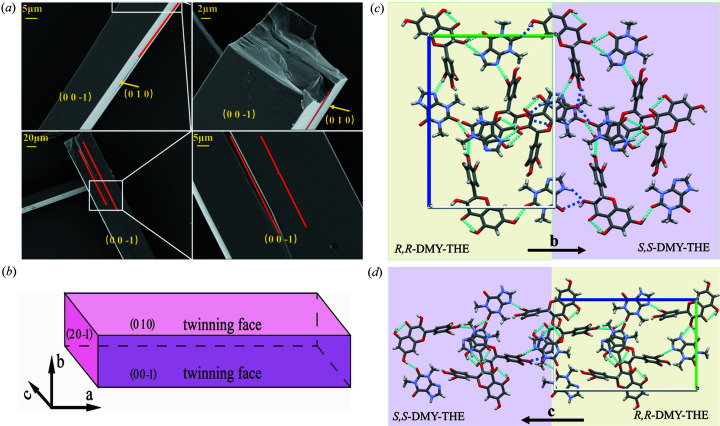
Twin boundary and indexed twin faces of the *Rac*-DMY–THE cocrystal.

The careful examination of the crystal morphology of the *Rac*-DMY–THE cocrystal reveals the presence of a twinning boundary that separates the crystal into several domains (Fig. 11 ▸). These domains are ordered and stacked parallel to the identical crystallographic orientation. The facet indexing results clarify the mutually epitaxial twin boundary that appears on the {010} and {001} sets. The width of the twins is roughly 5–20 µm, which is comparable to the coherent spot size of the utilized single-crystal X-ray beam in this study (∼10 µm). The mixing behavior of enantiomer domains within the crystal is likely on the micrometre-scale, neither as a molecular disorder in the molecular-scale mixing nor as an even large macroscopic crystal-scale mixing resemble that in the epitaxial conglomerate system.

The twin structure forms on the basal growing {010} and {001} surface, along the *a* axis, indicating a weaker interaction between the two enantiomers in the *b* and *c* directions. Fig. 6 ▸ suggests the rapid growth of DMY along the *a* axis is facilitated by strong hydrogen bonding interactions between *o*-phenyl­ene triols groups of the DMY molecule. The possible binding mode of the two enantiomeric molecules in *Rac*-DMY–THE cocrystals was illustrated in Fig. 11 ▸(*c*), with the twin boundaries highlighted. In both enantiomerically pure and racemic crystal structures of DMY–THE cocrystals, the THE and DMY molecules are linked by O—H⋯N and O—H⋯O hydrogen bonds along the *b* direction; in the *c* direction, the resorcinol groups *R*,*R*-DMY and *S*,*S*-DMY are joined together via an O—H⋯O hydrogen bond [dark-blue dashed line in Fig. 11 ▸(*d*)]. These hydrogen bonding interactions may be key factors in the early stages of nucleation and growth of enantiomorph cocrystals that lead to the formation of twin boundaries. However, note that the micrometre-scale mixing of enantiomer domains formed by epitaxial crystallization on *Rac*-DMY–THE cocrystals impedes the use of conventional chiral resolution methods, *e.g.* preferential crystallization.

### 3.4. Effect of molecular structure on the formation of the cocrystal solid solution of enantiomers

**Figure 12:**
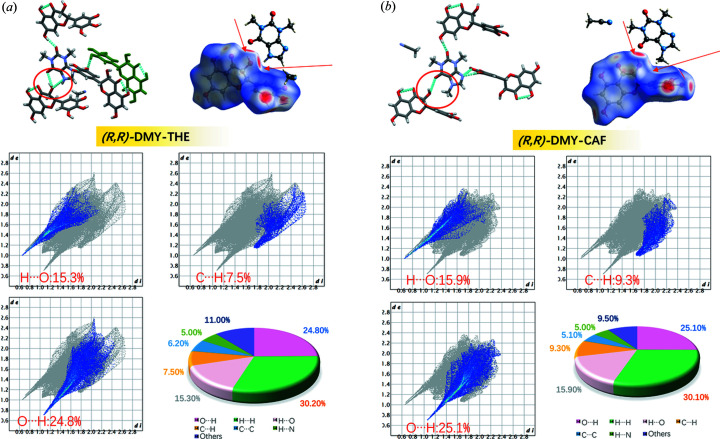
Comparisons of intermolecular interactions of *R*,*R*-DMY–THE and *R*,*R*-DMY–CAF cocrystals with HS and 2D fingerprint plots based on the *d*_norm_ value and the percentage contributions to the HS.

Two types of solid solution were found in our study in which the DMY–CAF cocrystal forms a type 2 chiral solid solution with molecular-scale mixing, whereas the DMY–THE cocrystal displays an inversion twin with micrometre-scale enantiomer mixing. The distinct mixing performance displaying several orders of magnitude difference is achieved only by the slight modification of the CAF coformer structure with the deletion of one methyl group on the five-membered ring of CAF (*i.e.* THE). Fig. 12 ▸ shows visually the difference in typical hydrogen bonding interactions of DMY and the coformer in a single enantiomeric cocrystal by HS analysis and 2D fingerprint plots. The red circles highlight the difference in the primary hydrogen-bonding mode, with THE forming hydrogen bonds through the carbonyl oxygen on the six-membered ring, N—H on the five-membered ring and the hydroxyl group of DMY. The HS in Fig. 12 ▸(*a*) displays two strong interactions (red regions indicated by arrows), whereas CAF forms only one strong O—H⋯O=C hydrogen bond between the carbonyl oxygen on the six-membered ring and the hydroxyl group of DMY [red region indicated by the arrow in Fig. 12 ▸(*b*)]. Besides, the B ring of DMY and the methyl H of CAF also form C—H⋯π weak interactions (blue region indicated by the arrow). 2D fingerprint plots reveal contributions of 15.3, 7.5 and 24.8% for H⋯O, C⋯H and O⋯H contacts in DMY–THE cocrystals, respectively, and 15.9, 9.3 and 25.1% for H⋯O, C⋯H and O⋯H in DMY–CAF cocrystals, respectively. The formation of C—H⋯π weak interactions between the B ring of DMY and the methyl of CAF may not satisfy the requirement of a tightly packed structure, and the optimal balance of interactions is achieved by forming two conformations for energy minimization and by forming pseudo-inversion centers, which allow for the (partial) replacement of chiral enantiomers in a pseudo-inversion center, resulting in molecular-level mixing. In contrast, there is only one set of molecular conformations in the asymmetric unit for the *Rac*-DMY–THE or *R*,*R*-DMY–THE cocrystal solid solution system, and the composition of the racemates is achieved by mixing crystal domains of differing chirality.

## 4. Conclusions

In this work, we investigated the enantioselectivity of chiral cocrystals of DMY enantiomers with two non-chiral coformers, CAF and THE, in solid-state chemistry. Although the mono-component enantiomer crystals rarely form a solid solution, we reported the two types of solid solutions constituting chiral cocrystals that display several orders of magnitude mixing performance of two enantiomers within the crystals. *Rac*-DMY–CAF co-crystals form a type 2 solid solution with an enantiomer mixed at the molecular scale, in which the single enantiomer adjusts its conformation to form a pseudo-inversion center to mimic the true symmetry center in the racemic crystal, allowing the formation of a solid solution. In contrast, *Rac*-DMY–THE cocrystals develop a racemic twin that shows enantiomer domains on the micrometre scale distributed in the crystal with the presence of racemic twins. The distinct enantioselectivity difference in two chiral cocrystal systems was attributed to the hydrogen-bonding donor–acceptor capacity of coformers where CAF co-crystallized with *R*,*R*-DMY can form two sets of conformations that satisfy pseudo-centric symmetry and realize the molecular-scale mixing of enantiomers. The stability of the cocrystal structure may be a subtle compromise between molecular energy and stacking efficiency. The degree of enantiomer mixing of cocrystals could impact their resolution method. Our findings could provide guidance for the separation of chiral racemic compounds with the cocrystallization method and the design of solid solution crystalline materials.

## Supplementary Material

Crystal structure: contains datablock(s) Rac-DMY-CAF, RR-DYM-CAF, Rac-DMY-THE-1, Rac-DMY-THE-2, Scalemic-DMY-THE-3, RR-DMY-THE. DOI: 10.1107/S2052252523000118/yc5040sup1.cif

Structure factors: contains datablock(s) RAC-DMY-CAF. DOI: 10.1107/S2052252523000118/yc5040Rac-DMY-CAFsup2.hkl

Structure factors: contains datablock(s) RR-DMY-CAF. DOI: 10.1107/S2052252523000118/yc5040RR-DMY-CAFsup3.hkl

Structure factors: contains datablock(s) RAC-DMY-THE-1. DOI: 10.1107/S2052252523000118/yc5040Rac-DMY-THE-1sup4.hkl

Structure factors: contains datablock(s) RAC-DMY-THE-2. DOI: 10.1107/S2052252523000118/yc5040Rac-DMY-THE-2sup5.hkl

Structure factors: contains datablock(s) Scalemic-DMY-THE-3. DOI: 10.1107/S2052252523000118/yc5040Scalemic-DMY-THE-3sup6.hkl

Structure factors: contains datablock(s) RR-DMY-THE. DOI: 10.1107/S2052252523000118/yc5040RR-DMY-THEsup7.hkl

Supporting figures and tables. DOI: 10.1107/S2052252523000118/yc5040sup8.pdf

CCDC references: 2178936, 2178963, 2178966, 2178975, 2179023, 2192790

## Figures and Tables

**Table 1:** Crystallographic data of DMY–CAF crystal structures

	*R*,*R*-DMY–CAF	*Rac*-DMY–CAF
Radiation type	Ga *K*α, λ = 1.34139 Å	Ga *K*α, λ = 1.34139 Å
Temperature (K)	193	193
Crystal system	Monoclinic	Monoclinic
Space group	*P*2_1_	*P*2_1_/*n*
*a* (Å)	14.650	14.5994
*b* (Å)	7.124	7.1223
*c* (Å)	24.455	24.470
α (°)	90	90
β (°)	101.688	101.455
γ (°)	90	90
*V* (Å^−3^)	2399.4	2493.7
ρ_calc_ (g cm^−3^)	1.476	1.48
*Z*, *Z*′, *Z*′′†	8, 2, 6	4, 1, 3
*R*_1_ [*I* > 2σ(*I*)]	0.0520	0.0904
*S*	1.041	1.074
Flack (μ)	0.1 (4)	–
Hooft (μ)	−0.0 (2)	–
Parson’q	−0.1 (3)	–
Disorder	–	0.57–0.43
CCDC No.	2192790	2178936

† *Z*′ is often defined as the number of formula units in the crystallographic unit cell divided by the number of independent general positions, but in this work *Z*′ = 1 represents only one set of THE, DMY and aceto­nitrile molecules in the asymmetric unit. The total number of chemical entities in the asymmetric unit has been referred to as *Z*″ by van Eijck & Kroon (2000 ▸).

**Table 2:** Crystallographic data of the DMY–THE cocrystal

	*R*,*R*-DMY–THE	*Rac*-DMY–THE-1	*Rac*-DMY–THE-2	*Scalemic*-DMY–THE-3
Diffraction source type	Rigaku Mo *K*α radiation	Rigaku Cu *K*α radiation	Rigaku Cu *K*α radiation	Rigaku Cu *K*α radiation
Temperature (K)	113	150	150	193
Crystal system	Orthorhombic	Orthorhombic	Orthorhombic	Orthorhombic
Space group	*P*2_1_2_1_2_1_	*P*2_1_2_1_2_1_	*P*2_1_2_1_2_1_	*P*2_1_2_1_2_1_
*a* (Å)	6.7292	6.7397	6.7429	6.7719
*b* (Å)	15.1840	15.2234	15.2146	15.2411
*c* (Å)	23.3897	23.4621	23.45083	23.471
α (°)	90	90	90	90
β (°)	90	90	90	90
γ (°)	90	90	90	90
*V* (Å^−3^)	2389.87	2407.24	2405.85	2422.5
ρ_calc_ (g cm^−3^)	1.505	1.494	1.495	1.485
*Z*, *Z*′, *Z*′′†	4, 1, 3	4, 1, 3	4, 1, 3	4, 1, 3
*R*_1_ [*I* > 2σ(*I*)]	0.0669	0.0378	0.0393	0.0355
*S*	1.056	1.062	1.138	1.071
Flack (μ)	–	0.49 (0.04)	0.54 (0.03)	0.4 (0.3)
Disorder	–	0.68–0.32	0.74–0.26	0.79–0.21
CCDC No.	2178963	2178966	2179023	2178975

† *Z*′ is often defined as the number of formula units in the crystallographic unit cell divided by the number of independent general positions, but in this work *Z*′ = 1 represents only one set of THE, DMY and aceto­nitrile molecules in the asymmetric unit. The total number of chemical entities in the asymmetric unit has been referred to as *Z*″ by Van Eijck & Kroon (2000 ▸).
