# Supplementary material for: Enantioselectivity of chiral di­hydro­myricetin in multicomponent solid solutions regulated by subtle structural mutation
Source: IUCrJ. 2023 Jan 21;10(Pt 2):164–76. doi: 10.1107/S2052252523000118 (PMC9980384; doi:10.1107/S2052252523000118)
Supplement: Supplementary file 8 [file m-10-00164-sup8.pdf]

IUCrJ

Volume 10 (2023)

Supporting information for article:

Enantioselectivity of Chiral Dihydromyricetin in Multicomponent Solid Solutions Regulated by Subtle Structural Mutation

Jie Sun, Yaoguo Wang, Weiwei Tang and Junbo Gong

S1. PXRD

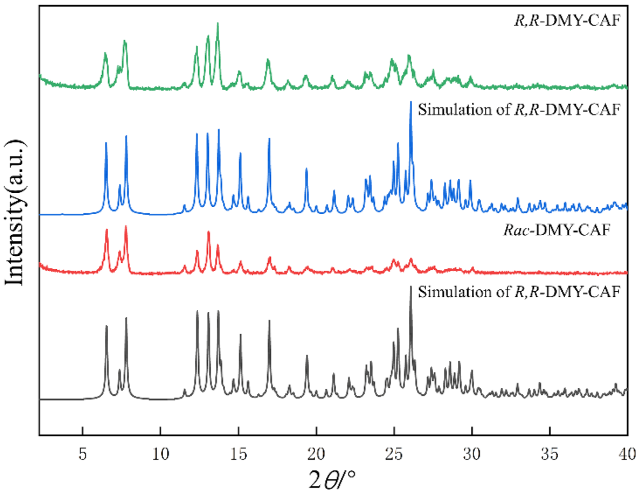

Figure S1 Experiment and simulation PXRD patterns of *R*, *R*-DMY-CAF and Rac-DMY-CAF cocrystals.

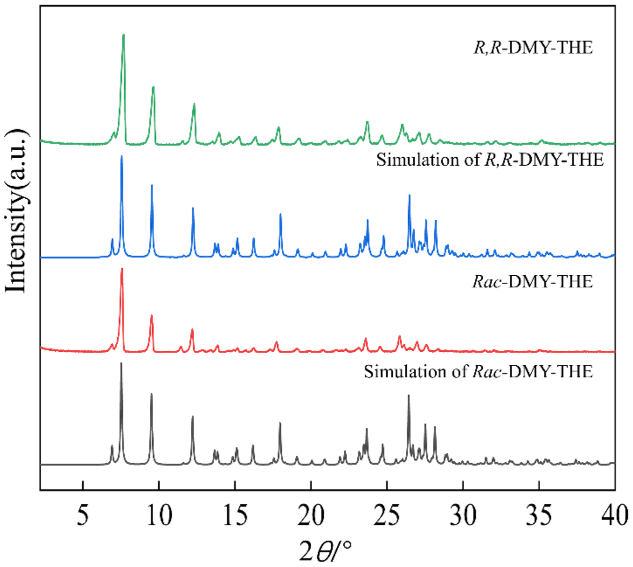

Figure S2 Experiment and simulation PXRD patterns of *R*, *R*-DMY-THA and Rac-DMY-THA cocrystals.

S2. Hot-stage microscopic measurements (HSM)

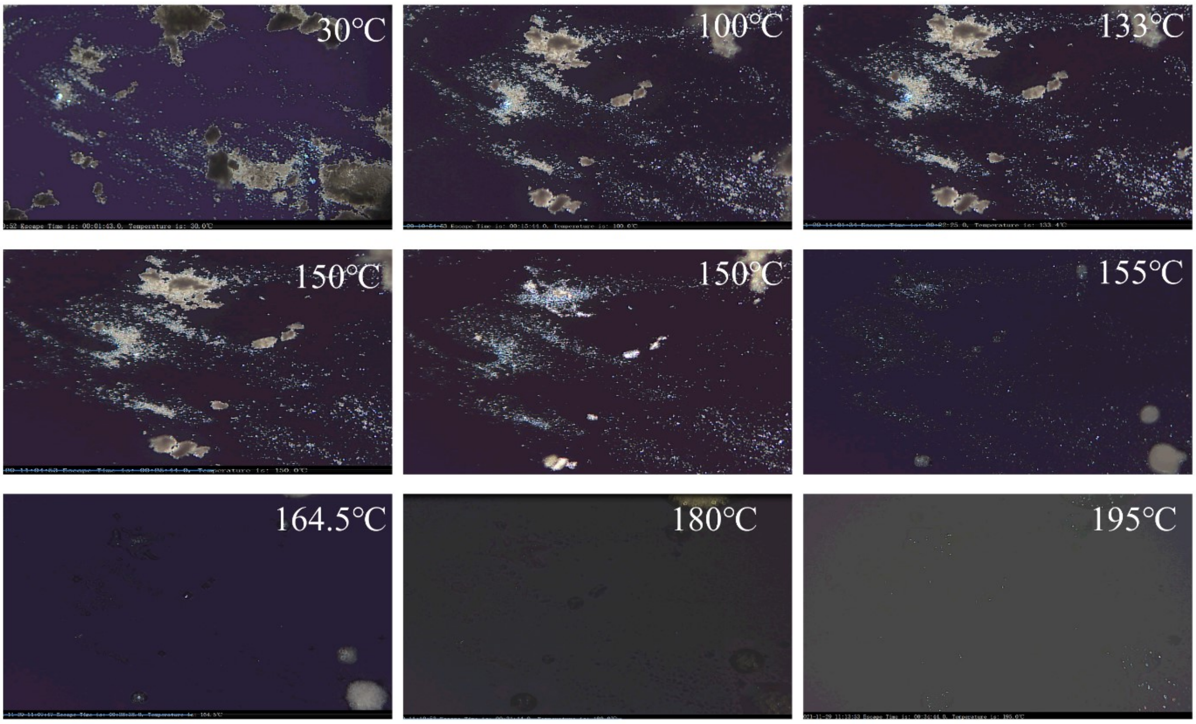

Figure S3 HSM of *Rac*-DMY-THE cocrystals.

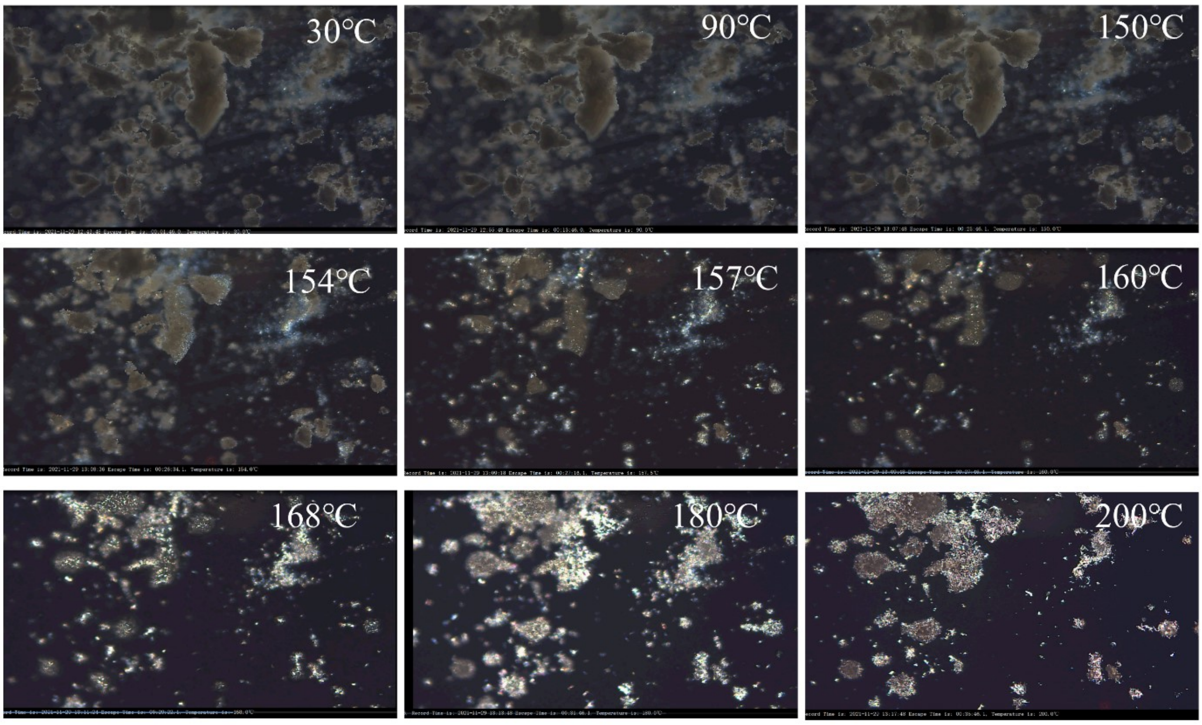

Figure S4 HSM of *R, R*-DMY-THE cocrystals.

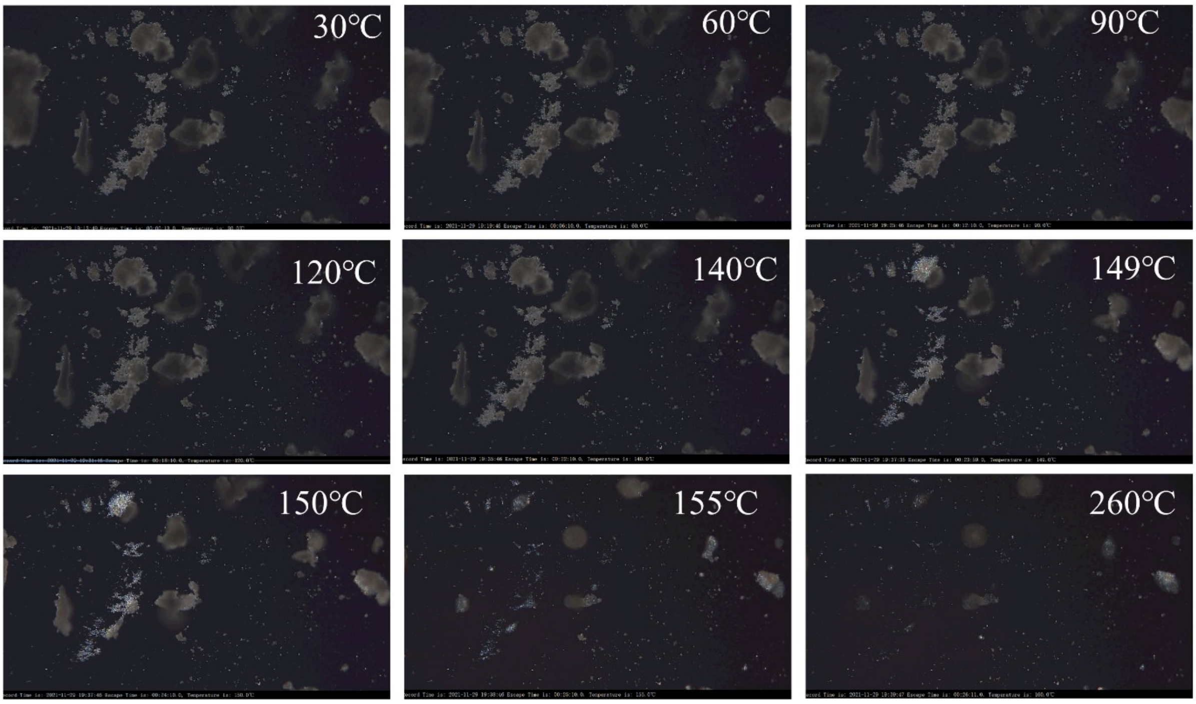

Figure S5 HSM of *Rac*-DMY-CAF cocrystals.

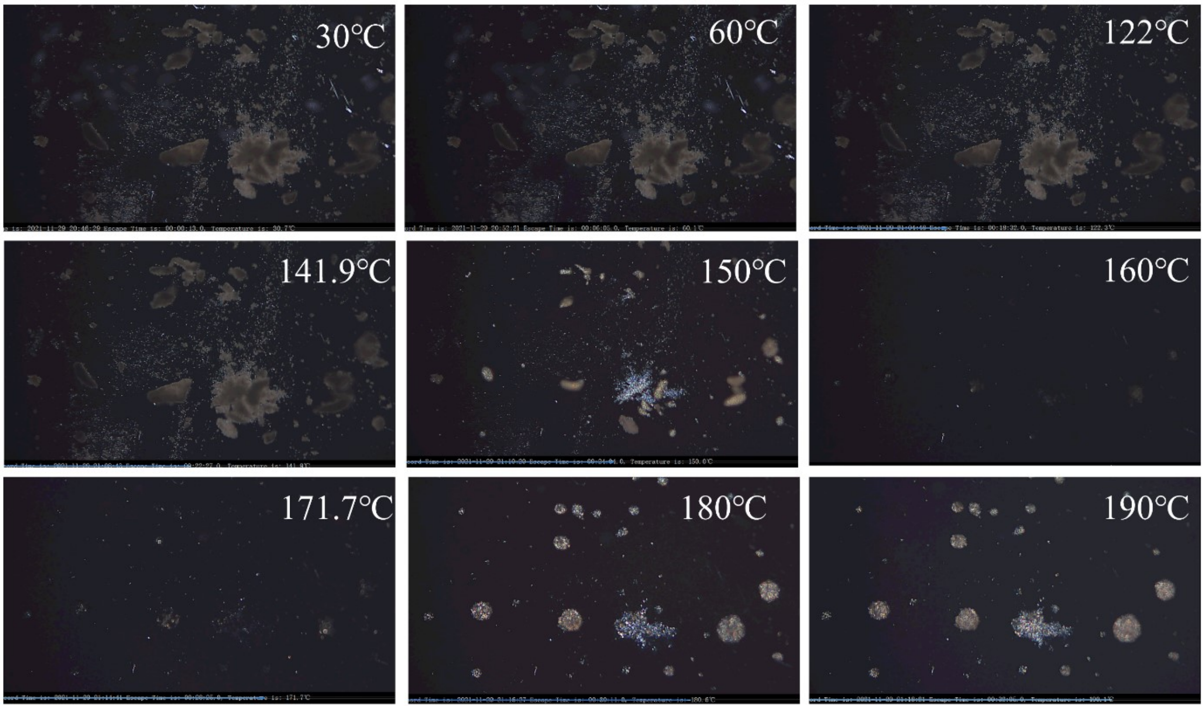

Figure S6 HSM of *R, R* -DMY-CAF cocrystals.

Table S1 Melting temperature (T_fus) values for DMY-THE cocrystals

$x_{R,R-DMY-THE}$	ee/%	T_{fus}/K
0.9816	96.32	150.29
0.9579	91.58	148
0.9008	80.15	147.18
0.8819	76.38	144.65
0.7636	52.71	142.96
0.7236	44.67	141.05
0.6673	33.46	142.31
0.6308	26.16	142.27
0.5947	18.95	141.3
0.5461	9.22	141.11
0.5345	6.90	142.26
0.5042	0.84	141.31

S3. Thin crystals peeled from the racemic crystal of DMY-THE.

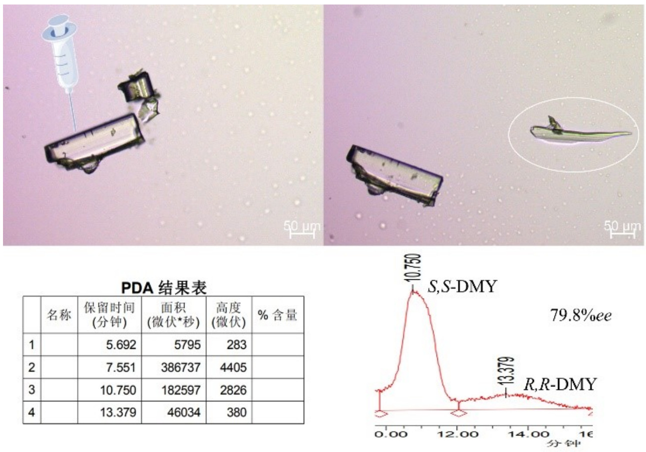

	名称	保留时间 (分钟)	面积 (微伏*秒)	高度 (微伏)	% 含量
1		5.692	5795	283	
2		7.551	386737	4405	
3		10.750	182597	2826	
4		13.379	46034	380	

Figure S7 HPLC of thin crystals peeled from the racemic crystal of DMY-THE

S4. Crystalline surface indexing.

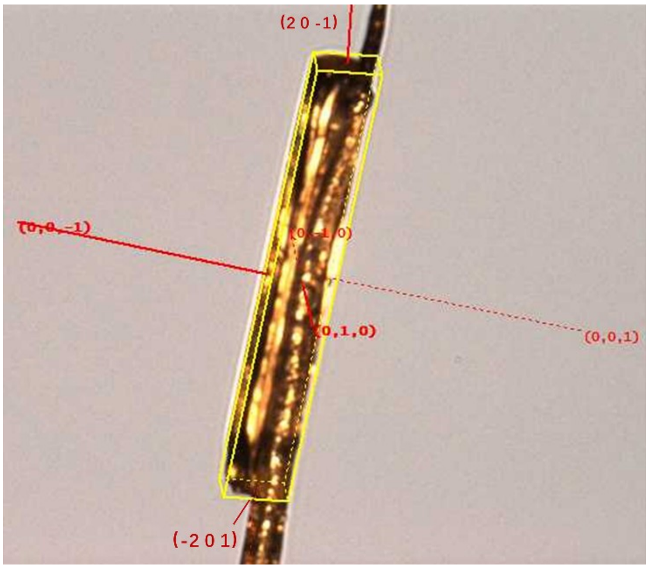

Figure S8 Crystalline surface indexing of Rac-DMY-THE

S5. HPLC of DMY-THE.

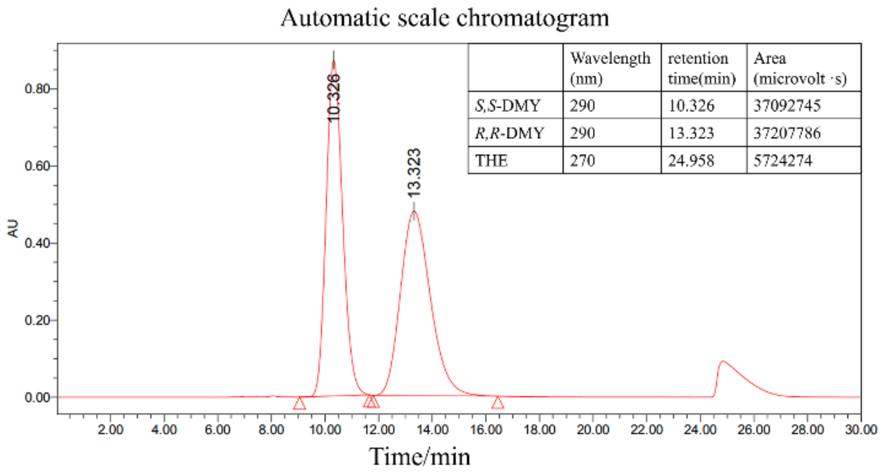

Figure S9 Automatic scale chromatogram of 0.003mol ml^{-1}Rac-DMY-CAF cocrystals

S6. Hydrogen Bonds for cocrystals in this work

Table S2 Hydrogen Bonds for Rac-DMY-CAF

D	H	A	d(D-H)/Å	d(H-A)/Å	d(D-A)/Å	D-H-A/°
O3	H3	O2	0.84	2.29	2.652(5)	106.0
O4	H4	O5	0.84	1.90	2.635(4)	144.8
O6	H6B	O1[1]	0.84	1.96	2.765(5)	159.1
O7	H7	N2[2]	0.84	1.90	2.716(5)	164.1
O8	H8	O3[3]	0.84	2.15	2.796(4)	133.0
O9	H9	O7[4]	0.84	1.93	2.672(5)	146.3

[1]-1/2+X,1/2-Y,1/2+Z; [2]3/2-X,1/2+Y,3/2-Z; [3]1/2+X,1/2-Y,1/2+Z; [4]+X,-1+Y,+Z

Table S3. Hydrogen Bonds for *R, R*-DMY-THE.

D	H	A	d(D-H)/Å	d(H-A)/Å	d(D-A)/Å	D-H-A/°
O1	H1	O101	0.84	1.80	2.639(4)	172.6
O2	H2	O4	0.84	1.89	2.631(5)	146.8
O5	H5	O92	0.84	2.00	2.720(4)	142.6
O6	H6	N1	0.84	1.95	2.764(5)	162.7
O7	H7	O13	0.84	2.27	2.834(5)	125.1
O8	H8	O64	0.84	1.93	2.720(5)	156.1
N2	H2B	O51	0.88	1.97	2.738(5)	145.3

[1]1-X,-1/2+Y,1/2-Z; [2]1-X,1/2+Y,1/2-Z; [3]1/2-X,1-Y,-1/2+Z; [4]-1+X,+Y,+Z
